# Resveratrol Inhibits Phenotype Modulation by Platelet Derived Growth Factor-bb in Rat Aortic Smooth Muscle Cells

**DOI:** 10.1155/2014/572430

**Published:** 2014-03-10

**Authors:** Mi Hee Lee, Byeong-Ju Kwon, Hyok Jin Seo, Kyeong Eun Yoo, Min Sung Kim, Min-Ah Koo, Jong-Chul Park

**Affiliations:** ^1^Cellbiocontrol Laboratory, Department of Medical Engineering, Yonsei University College of Medicine, 134 Shinchon-Dong, Seodaemun-Gu, Seoul 120-752, Republic of Korea; ^2^Brain Korea 21 PLUS Project for Medical Science, Yonsei University, 134 Shinchon-Dong, Seodaemun-Gu, Seoul 120-752, Republic of Korea

## Abstract

Dedifferentiated vascular smooth muscle cells (VSMCs) are phenotypically modulated from the contractile state to the active synthetic state in the vessel wall. In this study, we investigated the effects of resveratrol on phenotype modulation by dedifferentiation and the intracellular signal transduction pathways of platelet derived growth factor-bb (PDGF-bb) in rat aortic vascular smooth muscle cells (RAOSMCs). Treatment of RAOSMCs with resveratrol showed dose-dependent inhibition of PDGF-bb-stimulated proliferation. Resveratrol treatment inhibited this phenotype change and disassembly of actin filaments and maintained the expression of contractile phenotype-related proteins such as calponin and smooth muscle actin-alpha in comparison with only PDGF-bb stimulated RAOSMC. Although PDGF stimulation elicited strong and detectable Akt and mTOR phosphorylations lasting for several hours, Akt activation was much weaker when PDGF was used with resveratrol. In contrast, resveratrol only slightly inhibited phosphorylations of 42/44 MAPK and p38 MAPK. In conclusion, RAOSMC dedifferentiation, phenotype, and proliferation rate were inhibited by resveratrol via interruption of the balance of Akt, 42/44MAPK, and p38MAPK pathway activation stimulated by PDGF-bb.

## 1. Introduction

Vascular smooth muscle cells (VSMC) exhibit differentiated, biosynthetic, contractile roles in the media layer of mature blood vessels. However, VSMC dedifferentiation is induced in response to injury in a vessel, followed by phenotypic modulation toward a proliferative, migratory, and synthetic phenotype with extracellular matrix protein deposition, which contributes to intimal hyperplasia [1–3]. Differentiated VSMCs in normal vessels have a contractile phenotype, a spindle-like elongated morphology, and a smaller cell size. In contrast, dedifferentiated VSMCs in injured vessels have a synthetic phenotype, a hypertrophic appearance, hill and valley growth, and a relatively larger size. In addition to these morphological and functional alterations, the change in VSMCs from contractile to synthetic phenotype is controlled by SMC-specific molecular markers such as caldesmon, calponin, alpha-tropomysin, smooth muscle myosin heavy chain, SM22*α*, and smooth muscle alpha actin (*α*SMA) [4–6].

Platelet derived growth factor-bb (PDGF-bb) is one of the most potent mitogens and chemoattractants for VSMCs. PDGF-bb binds to the PDGF receptor (PDGFR)-*β* and utilizes the tyrosine kinase receptor signaling leading to generation of reactive oxygen species (ROS) and subsequently activates several intracellular signaling cascades, including the extracellular signal-regulated kinase (ERK) and p38 mitogen-activated protein kinase (MAPK) pathways and the phosphatidylinositol 3-kinase-Akt (PI3K-Akt) pathway. It has also been shown to stimulate VSMC dedifferentiation [7–9]. Akt is the major signal transducer in growth factor-mediated transcription and promotes cell survival by inhibiting apoptosis. Furthermore, the Akt pathway is the key trigger of mTOR signaling, and Akt-mediated phosphorylation is directly related to mTOR activation. The mTOR protein has been implicated in cardiovascular diseases and specifically in cardiac hypertrophy [10, 11].

Resveratrol (3,4′,5-trihydroxystilbene), a naturally occurring molecule known as a phytoalexin, is a polyphonic compound found in grapes and red wine. Resveratrol is also known to possess antioxidant, anti-inflammatory, antithrombotic, and antiproliferative effects. Additionally, various studies have shown that resveratrol inhibits the oxidation of low-density lipoprotein (oxLDL) and the early progression of atherosclerotic lesions and also protects cardiomyocytes against ischemia-reperfusion injury [12–14]. Although numerous studies have addressed the effects of red wine consumption on cardioprotection, alcohol contained in red wine interacts with resveratrol to elicit the desired effects. For that reason, several studies have demonstrated that both resveratrol supplement and dealcoholized red wine have physiological activity for cardiovascular protection [15, 16].

In this study, we investigated the effects of resveratrol on proliferation, phenotype modulation, and intracellular signal transduction pathways in PDGF-bb-induced rat aortic vascular smooth muscle cells (RAOSMCs). Our results demonstrate the inhibitory mechanism of resveratrol on phenotype modulation of PDGF-bb-stimulated RAOSMCs.

## 2. Materials and Methods

### 2.1. Cell Culture

Primary cultured rat aortic smooth muscle cells (RAOSMCs, Biobud, Seoul, Korea) were routinely maintained in Dulbecco's modified Eagle's medium (DMEM) (Gibco, Carlsbad, CA, USA) supplemented with 10% fetal bovine serum (Sigma, St. Louis, MO, USA) and a 1% antibiotic-antimycotic solution containing 10,000 units penicillin, 10 mg streptomycin, and 25 *μ*g/mL amphotericin B (Sigma) at 37°C in a humidified atmosphere of 5% CO_2_. For experiments, cells were used between passages 5 and 10.

### 2.2. Cell Stimulation by PDGF-bb and Treatment of Resveratrol

RAOSMCs were grown to 80–90% confluence and synchronized in serum-free DMEM medium for 48 h before experiments. Trans-resveratrol (Sigma) was dissolved in 50% dimethylsulphoxide (DMSO) (Sigma) for a stock solution of 100 mM and then diluted to desired concentrations with media prior to cell treatment. Cells were treated with various concentrations of resveratrol: 10~200 *μ*M in cell proliferation assay, 20 *μ*M in cell morphology analysis, and 100 *μ*M in western blotting on quiescent cells with or without 10 ng/mL PDGF-bb for designated times.

### 2.3. Cell Proliferation and DNA Synthesis

Cell viability was determined by MTT assay [reduction of 3-(4,5-dimethylthiazol-2-yl)-2,5-diphenyltetrazolium bromide to a purple formazan product, Sigma]. For the MTT assay, cells were incubated with 0.5 mg/mL MTT in the last 4 h of the culture period and tested at 37°C in the dark. The media were decanted, the produced formazan salts were dissolved in DMSO, and the absorbance was determined at 570 nm by an automatic microplate reader (Spectra Max 340, Molecular Devices Co., Sunnyvale, CA, USA). DNA synthesis was performed by a 5-bromo-2′-deoxyuridine (BrdU) incorporation assay (Roche Applied Science, Seoul, Korea). Briefly, BrdU-labeling solution was added to the cells, and cells were incubated for 2 h at 37°C. The labeling medium was then removed, and cells were incubated with fixation solution for 30 min at room temperature. After fixation of the cells, anti-BrdU-POD working solution was added, and cells were incubated for 90 min at room temperature. Then, the substrate solution was added, and the absorbance was measured at 370 nm with a 492 nm reference wavelength by an automatic microplate reader (Spectra Max 340, Molecular Devices Co.).

### 2.4. Immunofluorescence Assay

Cells were grown on coverslips to 50% confluence, serum-starved for 48 h, and then stimulated with or without 10 ng/mL PDGF-bb and 10 or 20 *μ*M resveratrol. Stimulated cells were fixed in 10% formalin solution and permeabilized with 0.5% Triton X-100 in phosphate buffer saline (PBS, pH 7.6). Then, cells were blocked with 5% bovine serum albumin (BSA, Sigma) and incubated with anti-smooth muscle actin-*α* (*α*SMA, Dako North America Inc., CA, USA) and anti-calponin (Santa Cruz Biotechnology Inc., Sana Cruz, CA, USA). Cells were then incubated with the secondary antibody, goat-anti-mouse IgG-conjugated Texas Red (Santa Cruz). Alexa 488-conjugated rhodamine phalloidin (5 U/mL, Invitrogen, Carlsbad, CA, USA) was used to visualize F-actin stress fibers, and nuclei were stained with Hoechst 33528. Coverslips were mounted with aqueous mounting medium (Dako Faramount, Dako North America Inc.), and images were evaluated using fluorescence microscope (Olympus, Tokyo, Japan) equipped with a DP-71 digital camera (Olympus).

### 2.5. Morphology Analysis

For morphology analysis, a cell plasma membrane was visualized by staining it with Texas Red C2-maleimide (5 U/mL, Invitrogen) and Hoechst 33258 (1 *μ*g/mL in PBS, Sigma). Images were captured on a fluorescence microscope (Olympus) equipped with a DP-71 digital camera (Olympus). Cell circularity and an area of 100 cells for each group were analyzed using ImageJ software (NIH, Bethesda, MD, USA). The circularity was measured to determine the morphological distribution between the contractile phenotype and the synthetic phenotype of RAOSMCs. Circularity was presented from 0 to 1, with values closer to 0 indicating spindle morphology and those closer to 1 indicating a circular phenotype [17, 18].

### 2.6. Western Blotting

After time-course stimulation with PDGF-bb, the cells were washed twice with cold PBS (10 mM, pH 7.4). Ice-cold RIPA lysis buffer (Santa Cruz Biotechnology) was added to the cells for 5 min. The cells were scraped, and the lysate was cleared by centrifugation at 14,000 ×g for 20 min at 4°C. The resultant supernatant (total cell lysate) was collected. The protein concentration was determined using a DC Bio-Rad assay kit (Bio-Rad Laboratories, Hercules, California, USA). For immunoblot analysis, proteins were separated by 10–15% SDS-PAGE and then electrotransferred onto a PVDF membrane. The membrane was blocked with blocking buffer (5% bovine serum albumin and 1% Tween-20 in 20 mM TBS, pH 7.6) for 1 h at room temperature and then probed overnight with antibodies to phospho-PDGFR-*β* (p-PDGFR-*β*, Tyr751), total PDGFR-*β*, phospho-MEK1/2 (p-MEK1/2, Ser217/221), total MEK1/2, phospho-p42/44MAPK (p-p42/44MAPK, Thr202/Tyr204), total p42/44MAPK, phospho-Akt (p-Akt, Ser473), total Akt, phospho-mTOR (p-mTOR, Ser2448), total mTOR, phospho-p38 MAPK (p-p38MAPK, Thr180/Tyr182), and total p38MAPK. Antibodies were purchased from Cell Signaling Technology (Danvers, MA, USA) and used at 1 : 1,000 dilutions. Detection of horseradish peroxidase-conjugated secondary antibody (i.e., anti-rabbit IgG (1 : 2,000) and antimouse IgG (1 : 2,000) from Santa Cruz Biotechnology Inc.) was accomplished using enhanced chemiluminescence of the ECL Plus detection kit (Amersham Biosciences, Buckinghamshire, England). The band intensity was quantified using ImageJ software (NIH) and relative fold exchanges averaged across the three experiments (normalized to phosphorylated forms and total forms) are shown below each band.

### 2.7. Statistical Analysis

All variables were tested in three independent cultures for each experiment. The results are reported as mean ± SD compared to nontreated controls. Statistical analysis was performed using a one-way ANOVA, followed by a Tukey's HSD test for multiple comparisons using SPSS software. A *P* value < 0.05 was considered statistically significant.

## 3. Results

### 3.1. Inhibitory Effect of Resveratrol on PDGF-bb-Induced Proliferation in RAOSMCs

**Figure 1:**
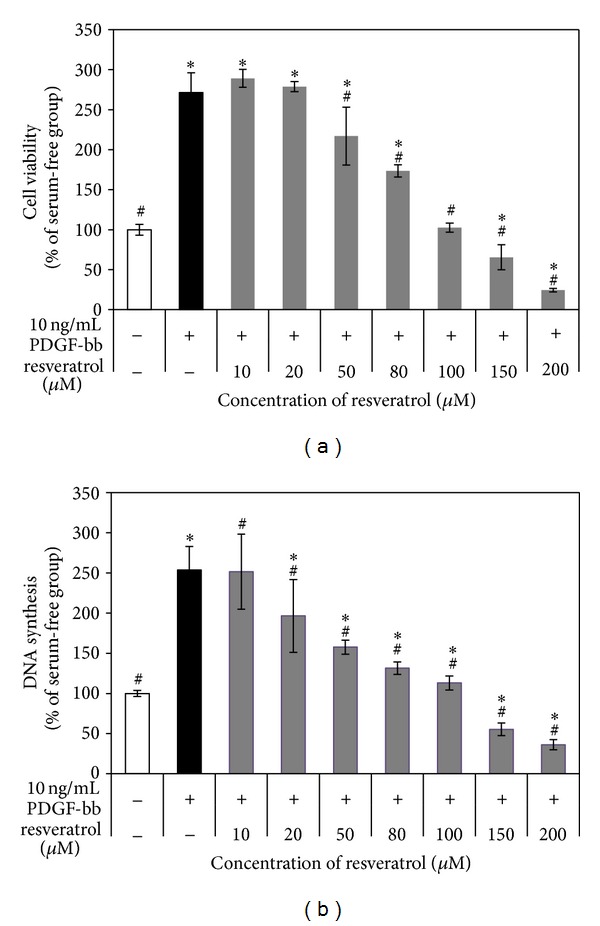
Antiproliferative activity of resveratrol in PDGF-bb-stimulated RAOSMCs. After 24 h of starvation with serum-free DMEM, cells were treated with 10 ng/mL PDGF-bb and increasing concentrations (10–200 *μ*M) of resveratrol for 48 h. (a) The effect of resveratrol growth inhibition on PDGF-bb-stimulated RAOSMCs. Cell viability was detected using the MTT assay. (b) The effect of resveratrol on PDGF-bb-induced DNA synthesis in RAOSMCs. DNA synthesis was detected using the BrdU incorporation assay. *P < 0.05 compared with nonstimulated controls; ^#^ *P* < 0.05 compared with 10 ng/mL PDGF-bb-stimulated controls.

To assess whether resveratrol inhibited PDGF-bb-stimulated RAOSMC proliferation, serum-starved RAOSMCs were incubated with 10 ng/mL PDGF-bb and increasing concentrations of resveratrol for 48 h. Treatment with 10 ng/mL PDGF-bb induced proliferation of RAOSMCs in comparison with nonstimulated cells. However, the presence of resveratrol resulted in significant (*P* < 0.05) dose-dependent decreases in cell growth (Figure 1(a)). When cells were treated with increasing concentrations of resveratrol, a significant (*P* < 0.05) dose-dependent reduction in cell growth was observed starting at 50 *μ*M. The level of DNA synthesis was also measured by cell proliferation assay. Stimulation of RAOSMCs with 10 ng/mL PDGF-bb caused a significant increase in the DNA amount, and resveratrol significantly inhibited this increase in a concentration-dependent manner (Figure 1(b)). Furthermore, increases in cell viability and DNA synthesis induced by PDGF-bb stimulation were completely suppressed in cells treated with concentrations of resveratrol greater than 100 *μ*M. These results suggest that resveratrol exerts a potent antiproliferative effect on PDGF-bb-stimulated RAOSMC proliferation.

### 3.2. Inhibitory Effect of Resveratrol on PDGF-bb-Stimulated RAOSMC Morphology and Phenotype

To define phenotype exchange in PDGF stimulation, we assessed RAOSMC phenotype and morphology using immunofluorescence and immunocytochemical staining with antibodies against *α*SMA and calponin. RAOSMCs were grown on glass cover slips, starved of serum for 48 h, and stimulated in the presence and absence of PDGF-bb and resveratrol for 24 h.

**Figure 2:**
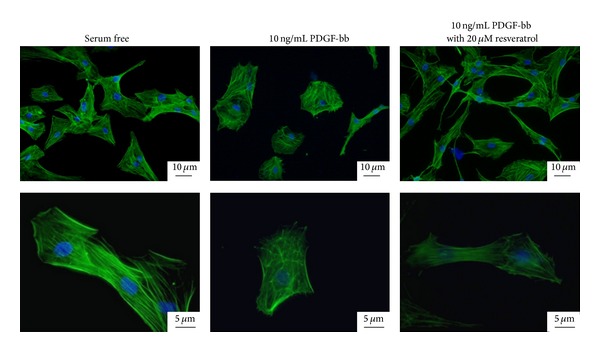
Arrangement of F-actin filaments of RAOSMCs with or without 10 ng/mL PDGF-bb stimulation and 20 *μ*M resveratrol. Nuclei were stained blue with Hoechst 33528, and F-actin was green due to alexa (388)-rhodamine phalloidin. Images in the upper and low panels were obtained at ×400 and ×1000 original magnifications, respectively. The micrographs shown in this figure are representative of three independent experiments with similar results.

RAOSMCs exhibited elongation and spindle morphology during prolonged serum deprivation. As shown in Figure 2, RAOSMCs serum-starved for 48 hr revealed an aligned arrangement of actin filaments with an organized cytoskeleton network. In contrast, PDGF-bb-stimulated RAOSMCs showed disassembled distribution and aggregation around the perinuclear region of actin filaments without clear filamentous organization. However, RAOSMCs treated with resveratrol maintained spindle-like shapes and organization of the actin filaments by inhibiting the effects of PDGF-bb-stimulation.

**Figure 3:**
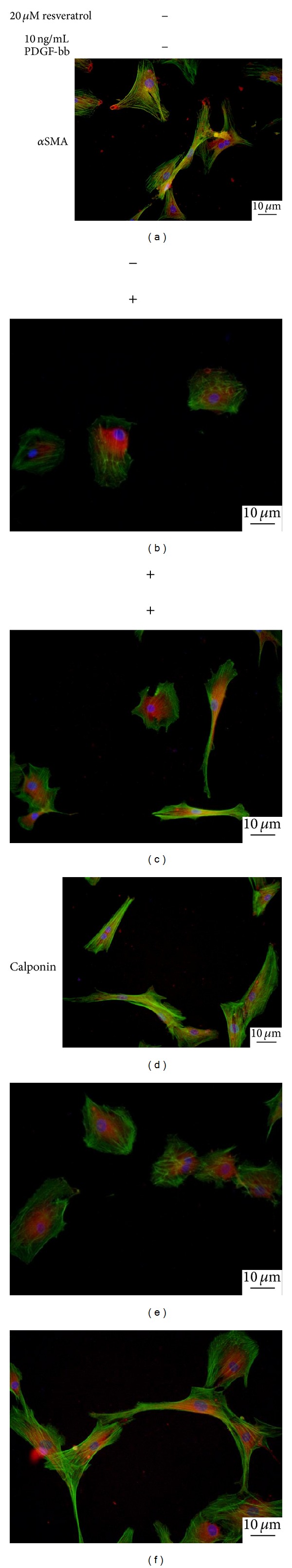
Characterization of morphology modulation by resveratrol on PDGF-bb-stimulated RAOSMCs. Cells were incubated in serum-free media (a, d), 10 ng/mL PDGF-bb (b, e), or 20 *μ*M resveratrol with 10 ng/mL PDGF-bb (c, f). Nuclei were stained blue with Hoechst 33528, *α*SMA (a~c) and calponin (d~f) are red, and F-actin is green due to alexa (388)-rhodamine phalloidin. The micrographs (magnification, ×100) shown in this figure are representative of three independent experiments with similar results.

**Figure 4:**
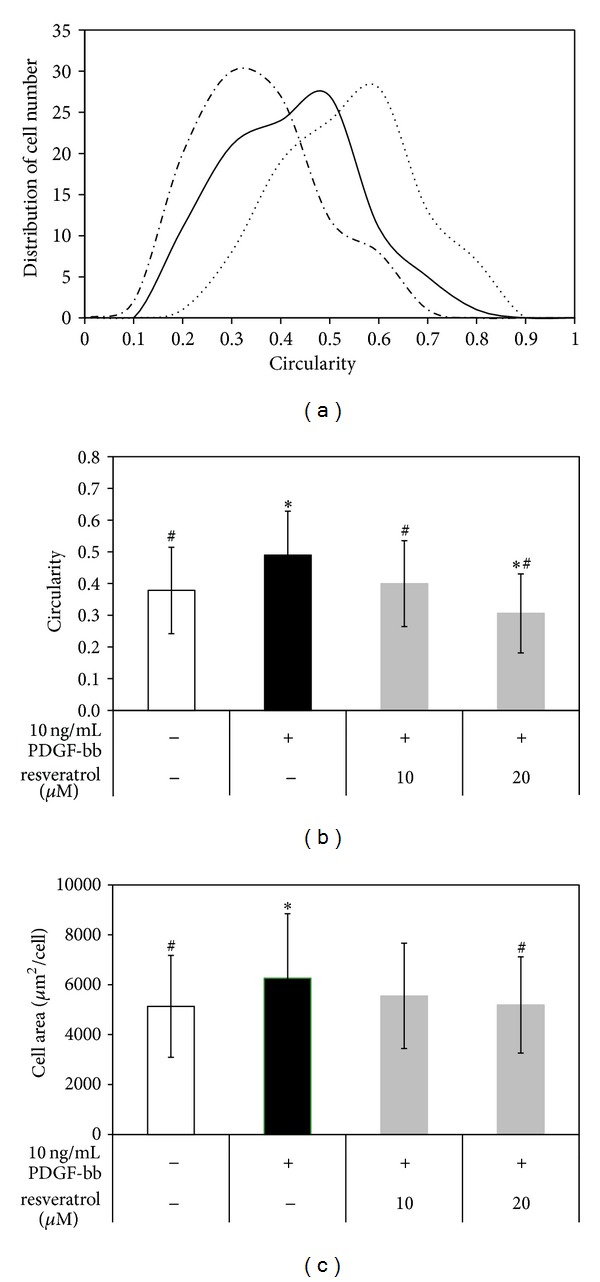
Morphology modulation by resveratrol in PDGF-bb-stimulated RAOSMCs. (a) The distribution of circularity ranged from 0 to 1, for linear to circular, respectively. (—in serum free, —•— in 10 ng/mL PDGF-bb, and ••••• in 10 ng/mL PDGF-bb with 20 *μ*M resveratrol). (b) The average circularity ±SD was obtained from 100 single cells per each type. *P < 0.05 compared with nonstimulated control; ^#^ *P* < 0.05 compared with 10 ng/mL PDGF-bb-stimulated control. (c) The average area (*μ*m^2^/cell) ± SD was obtained from 100 single cells of each type. *P < 0.05 compared with nonstimulated control; ^#^ *P* < 0.05 compared with 10 ng/mL PDGF-bb-stimulated control.

Therefore, cells were examined for accumulation of the contractile-related proteins, *α*SMA (Figure 3(a)) and calponin (Figure 3(d)), in clear actin filament organization. When RAOSMCs were stimulated with PDGF-bb, cells were observed to undergo morphological changes from spindle-shaped to polygonal, and relatively low levels of *α*SMA (Figure 3(b)) and calponin were noted (Figure 3(e)) with disassembled distribution of actin filaments in the cytosol. However, treatment with 20 *μ*M resveratrol inhibited the morphological changes. Furthermore, when cells were treated with resveratrol, the actin cytoskeleton maintained parallel actin filaments by forming a complex with contractile phenotype-related proteins, including *α*SMA (Figure 3(c)) and calponin (Figure 3(f)). To determine the morphological distribution of the cells, circularity was measured by ImageJ analysis. Compared to serum-starved cells, PDGF-bb-stimulated cells showed a greater distribution in circularity, whereas cells treated with resveratrol exhibited lower circularity (Figure 4(a)). The average circularity of PDGF-bb-stimulated cells was significantly higher than that of nontreated cells. However, resveratrol-treated cells stimulated with PDGF-bb were not significantly different from the nontreated cells (Figure 4(b)). Therefore, PDGF-bb-stimulated RAOSMCs had a greater area compared with serum-starved RAOSMCs. Resveratrol inhibited the change in area stimulated by PDGF-bb (Figure 4(c)).

### 3.3. Inhibitory Mechanism of Dedifferentiation on PDGF-bb-Stimulated RAOSMCs by Resveratrol

Treatment of RAOSMCs with resveratrol significantly inhibited PDGF-bb-stimulated proliferation in a dose-dependent manner. Furthermore, treatment of cells with 100 *μ*M resveratrol almost completely inhibited the growth of ROASMCs, as shown by DNA synthesis assay and MTT assay.

To define the effects of resveratrol on signaling pathways involved in PDGF-bb-stimulated dedifferentiation, serum-starved cells were stimulated with 10 ng/mL PDGF-bb in the absence or presence of resveratrol for specified times. Then, we analyzed the activation of p42/44MAPK, p38MAPK, and Akt, downstream effectors of PDGF-bb-induced signaling, by Western blotting.

**Figure 5:**
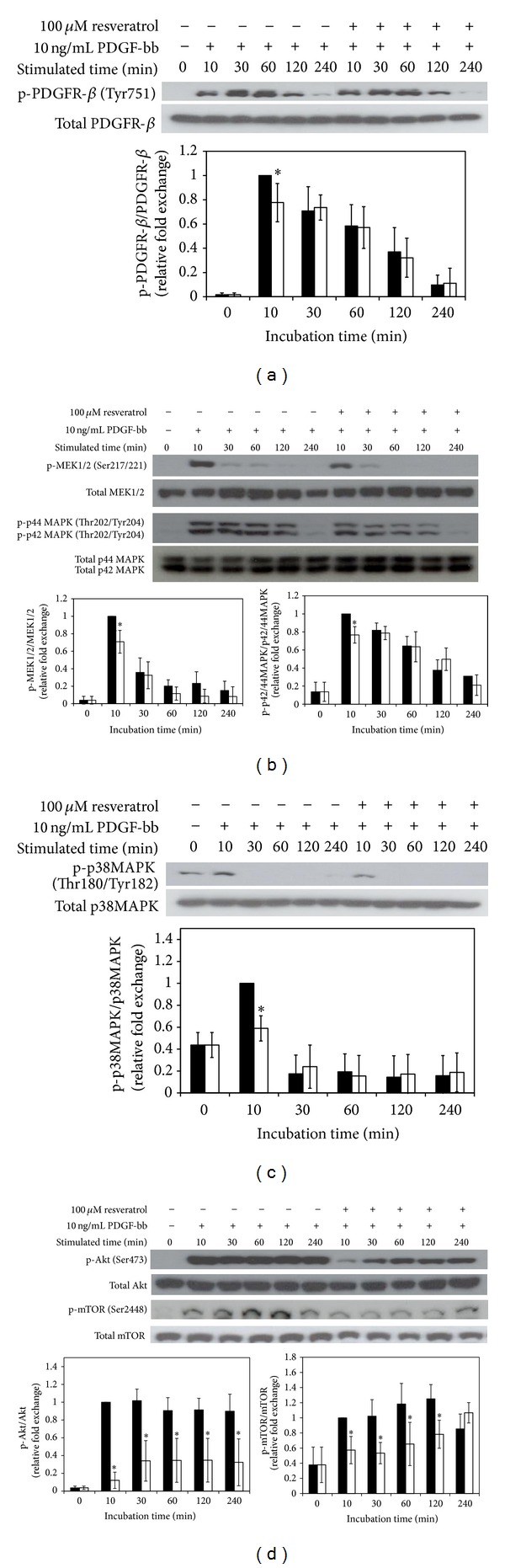
The effects of resveratrol on modulation of PDGF-bb-stimulated signaling pathways in RAOSMCs. RAOSMCs starved of serum were stimulated with 10 ng/mL PDGF-bb and 100 *μ*M resveratrol for the indicated times (10 m, 30 m, 1 h, 2 h, and 4 h) and lysed. Lysates were immunoblotted with antibodies. After densitometric quantification using the ImageJ program, data were expressed each as the mean ± SD from three independent experiments. Black bar indicates expression by PDGF-bb stimulation. White bar indicates expression by PDGF-bb stimulation with EGCG. (a) The expression of phospho-PDGFR-*β* in a time-dependent manner. The band intensities were normalized to PDGFR-*β* expression. (b) The time-dependent expressions of phospho-MEK1/2 and phospho-p42/44MAPK. The band intensities were normalized to MEK1/2 and p42/44MAPK expression. (c) The time-dependent expression of phospho-p38 MAPK. The band intensities were normalized to p38 MAPK expression. (d) The time-dependent expression of phospho-Akt and phospho-mTOR. The band intensities were normalized to Akt and mTOR expression.

Addition of 10 ng/mL PDGF-bb to serum-starved cells led to PDGFR-*β* phosphorylation that peaked within 10 min of stimulation and returnedto baseline level after 2~4 h, with similar results achieved in atleast three independent experiments (Figure 5(a)). However, resveratrol treatment inhibited PDGFR-*β* phosphorylation induced byPDGF-bb at only 10 min, while no inhibition of PDGFR-*β* phosphorylation was observed during incubations longer than 30 min.

In a similar manner, PDGF-bb stimulated the phosphorylations of downstream effectors such as MEK1/2, p42/44MAPK (Figure 5(b)), and p38 (Figure 5(c)), and resveratrol only slightly inhibited the PDGF-bb-induced phosphorylations of MEK1/2, p42/44MAPK, and p38MAPK in a time-dependent manner. However, as shown in Figure 5(d), while PDGF stimulation elicited strong and detectable signals for Akt and mTOR phosphorylations for several hours, resveratrol treatment clearly inhibited the PDGF-mediated phosphorylations of Akt and mTOR, a downstream effector dependent on Akt.

## 4. Discussion

Changes of the differentiated VSMC play a critical role in cardiovascular diseases, such as atherosclerosis, hypertension, asthma, and vascular aneurisms. Identification of dedifferentiated VSMCs was based on morphological criteria that met the terms for “phenotypic modulation” or “phenotypic switching” in functional and structural properties. A phenotypic switch from a contractile to synthetic phenotype accompanies the proliferation and migration of cells [19, 20].

PDGF, a key mediator in the proliferation of VSMCs, plays an important role in the pathogenesis of various vascular disorders. It has already been reported that PDGF-bb is implicated in intracellular ROS generation and VSMC growth [7, 21]. Furthermore, the signaling pathways affected by PDGF are so similar to that activated by oxidative stress [22, 23]. PDGF-bb represses the characteristic VSMC gene expression by activating extracellular signal-regulated kinase 1/2-mitogen activated protein kinase (ERK1/2-MAPK), p38 MAPK, and Akt pathways in cultured VSMCs [24, 25].

This study reports that PDGF-bb treatment causes phenotypic changes typical of dedifferentiation in RAOSMCs by activating p42/44 MAPK, p38 MAPK, and Akt pathways. In our experimental system, resveratrol inhibited the proliferation of PDGF-bb-stimulated RAOSMCs. The contractile morphology and spindle phenotype of the cells were also preserved by resveratrol treatment. Therefore, markers of VSMC differentiation, such as *α*SMA and calponin, were not inhibited by resveratrol treatment. In other words, resveratrol might prevent morphologic changes from contractile to synthetic phenotype. VSMCs in mature animal vessels exhibit a contractile phenotype (differentiated state) and express multiple contractile proteins, including *α*SMA, SM22*α*, and SM-MHC [26, 27]. Calponin and *α*SMA are characterized in detail as F-actin binding components of smooth muscle thin filaments, and they control actin-based cellular processes by regulating the stability of the actin cytoskeleton [6, 28].

The investigation of signal transduction pathways induced by PDGF-bb showed that resveratrol can inhibit phosphorylation of PDGFR-*β*, 42/44 MAPK, Akt, and p38MAPK. In particular, Akt/mTOR phosphorylation was preferentially inhibited in PDGF-bb-induced cells treated with resveratrol. It is known that VSMC phenotype is determined by changes in the balance of activation between the Akt pathway, the ERK, and p38MAPK pathways. Therefore, the Akt pathway plays a vital role in maintaining the differentiated phenotype [29].

The phosphoinositol-Akt-mammalian target of the rapamycin-p70S6 kinase (PIK/Akt/mTOR/p70S6K) pathway regulates cell growth and cell differentiation in response to nutrients, growth factors, and cytokines [30, 31]. Pharmacological inhibition with rapamycin was shown to induce contractile morphology, SM2-MHC, and calponin, protein reduction, and collagen synthesis in cultures of synthetic phenotype VSMCs by regulation of the mTOR/p70 S6 K1 pathway [30]. Previous studies have shown that mTOR activation induced SMC proliferation and required the activation of the signaling cascade PI3K/PDK1/Akt, as assessed by the effect of the PI3K inhibitors wortmannin and Ly294002, which block PDK [10, 11, 32]. Thus, resveratrol inhibited SMC phenotypic modulation by changing the balance between the Akt and MAPK pathways via hindering PDGF-bb-induced Akt pathways, but not the ERK and p38MAPK pathways. Furthermore, inhibition of Akt/mTOR pathways by resveratrol in SMC affected not only DNA synthesis, but also expression of phenotype-related proteins. Acute or chronic administration of plant polyphenols in patients has been found to result in the vasoprotective, antiangiogenic, antiatherogenic, vasorelaxant, and antihypertensive effects [33]. Several reports demonstrate resveratrol's efficacy in inhibiting VSMC proliferation [34, 35]. Furthermore, it has been shown that resveratrol specifically blocks the PI3K/PDK1/Akt pathway, thereby inhibiting oxLDL-induced SMC proliferation [14].

In this study, we focused on the effect of resveratrol on phenotypic modulation of RAOSMCs following stimulation with PDGF-bb. From a general point of view, resveratrol exhibits various potentially inhibitory properties on dedifferentiation, including an antiproliferative effect and an ability to modulate important growth signaling pathways.

## 5. Conclusion

In this study, we investigated the effect of resveratrol on dedifferentiation of RAOSMCs induced by PDGF-bb. Resveratrol inhibited dedifferentiation, phenotypic alterations, and proliferation rate stimulated by PDGF-bb in RAOSMC. In conclusion, our results indicate that this effect was probably mediated via a differential regulation of the balance between Akt, 42/44MAPK, and p38MAPK pathway activations stimulated by PDGF-bb at least in part for the effect of resveratrol. This result suggests that resveratrol may be an inhibitor of the phenotype modulation occurring in arterial stenosis and in postangioplasty restenosis following vascular injury.
